# Phospholipid Membrane Protection by Sugar Molecules during Dehydration—Insights into Molecular Mechanisms Using Scattering Techniques

**DOI:** 10.3390/ijms14048148

**Published:** 2013-04-12

**Authors:** Christopher J. Garvey, Thomas Lenné, Karen L. Koster, Ben Kent, Gary Bryant

**Affiliations:** 1 Australian Nuclear Science and Technology Organisation, Locked Bag 2001, Kirrawee DC NSW 2232, Australia; E-Mail: benk@ansto.gov.au; 2 Research School of Biological Sciences, the Australian National University, Canberra, ACT 0200, Australia; E-Mail: thomas.lenne@atsb.gov.au; 3 Department of Biology, The University of South Dakota, Vermillion, SD 57069, USA; E-Mail: karen.koster@usd.edu; 4 School of Applied Sciences, RMIT University, Melbourne, VIC 3001, Australia; E-Mail: gary.bryant@rmit.edu.au

**Keywords:** cryobiology, anhydrobiology, X-ray scattering, contrast variation small angle neutron scattering, membranes, phospholipids, sugars

## Abstract

Scattering techniques have played a key role in our understanding of the structure and function of phospholipid membranes. These techniques have been applied widely to study how different molecules (e.g., cholesterol) can affect phospholipid membrane structure. However, there has been much less attention paid to the effects of molecules that remain in the aqueous phase. One important example is the role played by small solutes, particularly sugars, in protecting phospholipid membranes during drying or slow freezing. In this paper, we present new results and a general methodology, which illustrate how contrast variation small angle neutron scattering (SANS) and synchrotron-based X-ray scattering (small angle (SAXS) and wide angle (WAXS)) can be used to quantitatively understand the interactions between solutes and phospholipids. Specifically, we show the assignment of lipid phases with synchrotron SAXS and explain how SANS reveals the exclusion of sugars from the aqueous region in the particular example of hexagonal II phases formed by phospholipids.

## 1. Introduction

Cell membranes exist as selective barriers between the cell cytoplasm, various intracellular compartments and the extracellular world. They may facilitate transport or act as a variable permeability barrier for solutes and solvent (water) molecules. The transport properties of the membrane depend on the proteins that mediate the movement of most solutes and on the physical properties of the membrane lipids forming the bilayer in which the proteins are embedded [1,2]. Maintaining the correct functioning of this permeability barrier is of critical importance to the viability of the cell. Cellular dehydration (caused by freezing and/or dry environments) causes changes in membrane lipid organization, which, in turn, bring about the loss of the normal semi-permeability of the membrane and, thus, death of the cell [3–6].

Generally, the transport of solutes and macromolecules across the cell membrane is much slower than that of water, and it is the water distribution that responds most quickly to changing environmental conditions, such as dehydration—depending on the species and tissue, some of the water transport occurs through the lipids of the cell membrane, while more rapid diffusion occurs through specific water channels, called aquaporins [3,5]. Thus in slow drying conditions and at temperatures above the formation of the glassy state where molecular mobility is abruptly arrested, one can assume that water potentials will come to equilibrium through water diffusion and that solutes will not redistribute across the membrane appreciably during the drying process [4]. The effects of slow cooling are equivalent—when ice forms in the extracellular solution the concentration of extracellular solutes—is increased, and because the membrane is relatively permeable to water, water may be drawn out of the cell much more quickly than solutes may be transported in. As further cooling occurs, the volume fraction of ice increases, further increasing the solute concentration in the non-frozen fraction, and more water is drawn out of the cell. Thus, the net effect of freezing on slow timescales is to dehydrate and contract the volume of the intracellular solution and is, in fact, similar to the effects of drying [6,7]. Recent work has also suggested that the effects of sugars on membranes are very strongly concentration-dependent [8], with sugar lipid interactions at very low sugar concentrations, but exclusion from the membrane surface at higher concentrations [9].

### 1.1. Membrane Protection by Small Solutes

High concentrations of small sugar molecules can help maintain the viability of cells during slow freezing or drying [7,10–13]. Similarly, the integrity of model membranes may be maintained by the presence of sugars during changes in hydration caused by freezing or thawing [14,15]. A much-cited explanation of this effect proposes a specific interaction between lipid head groups and sugar molecules [16]. The interaction involves the replacement of water molecules at the lipid head groups by sugar molecules; thus, this model is termed the water replacement hypothesis (WRH) [17,18]. The proposed interaction is very specific, and the WRH is heavily reliant on the specificity of certain small solutes, such as trehalose and sucrose, as protectants [16]. In addition, the WRH is only a qualitative model. An alternate explanation is the hydration forces explanation (HFE) [6,19], which frames the protective mechanism of sugars in terms of the modulation of the interaction between membranes as they come into increasingly close apposition as cell volumes contract during drying or freezing. The effects of vitrification (the glass transition) are also important at very low temperatures and/or hydrations [11,20,21], and the effect of glass formation on membranes has been quantitatively explained previously using the HFE [22]. It is now well established that the HFE can quantitatively explain the membrane protective effects of solutes at low and intermediate hydrations, although specific interactions (as proposed by the WRH) may be important in completely dry systems.

### 1.2. Hydration Forces Explanation

At the heart of the HFE is the loss of cell volume accompanying the loss of cell water, bringing membrane bilayers into close apposition, where they experience short-range repulsive hydration interactions, which can damage the membrane [23–26]. This model for the interactions between membrane bilayers has been experimentally verified by direct measurement of the forces between model membranes brought into close approach. Short-range forces between bilayers have been measured using a variety of experimental techniques, and the short-range repulsive hydration force has been clearly identified [27–30]. Sugars have been found to modulate this interaction, reducing the short-range repulsive hydration interaction [31]. As the membranes come into close repulsive apposition, the short-range hydration interaction becomes dominant, inducing a lateral compressive stress in the membrane. This compressive stress is responsible for transitions from the fluid lamellar phase (associated with normal membrane function) to other potentially deleterious phases, such as the gel and inverse hexagonal phases. The effects of the lateral compression in the membrane include demixing of membrane constituents, such as proteins and lipids, as well as phase transitions in the membrane [6,19,32]. The effect of sugars is to reduce the hydration force between membranes and, thus, the lateral compression in the plane of the membrane.

### 1.3. Phase Behavior of Phospholipids at Low Hydration

**Figure 1:**
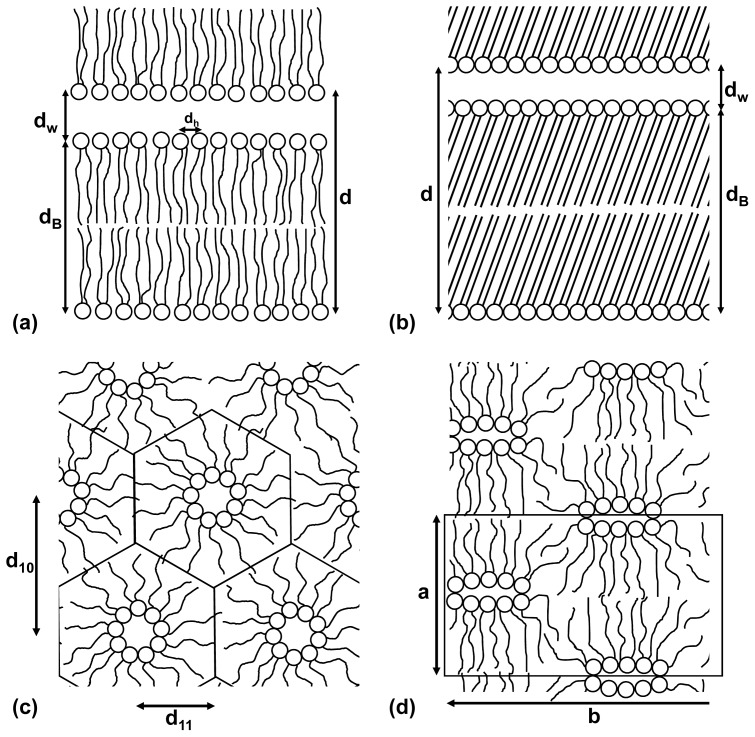
The lipid phases involved in freezing or desiccation-induced cellular damage: (**a**) the fluid lamellar phase consists of alternating layers of lipid bilayers (thickness, *d*_B_) and water (thickness, *d*_w_) and separation between head groups, *d*_h_. In the fluid lamellar phase, the tail chains are packed rather randomly in the hydrophobic phase; (**b**) the gel phase is very similar in geometry, with the difference being a closer packing of head groups and extended frozen lipid chains; (**c**) the hexagonal phase causes loss of bilayer structure and is characterized by a hexagonal symmetry with two characteristic repeat distances. Each hexagon has at its center a circular channel of water projecting out of the page surface; (**d**) the ribbon phase, where the unit cell is again characterized by two characterized by repeat distances. A ribbon-like channel formed by lipid head groups projects out of the page.

In order to examine these effects, we have undertaken a range of scattering studies on model systems consisting of phospholipids and simple sugars at a range of hydration levels. While real membranes are complex mixes of lipids and macromolecules, in order to make the problem tractable from a theoretical and experimental viewpoint, our work has focused on model lipid systems, which exhibit the same general trends as membranes. Lipids may exist in a number of different phases, depending on hydration and temperature [33,34], and several of these are shown schematically in Figure 1. Under normal physiological conditions (full hydration), phospholipid membranes exist primarily in the fluid lamellar phase (Figure 1a). As water is removed at constant temperature, the phospholipids can undergo a transition to the gel phase (Figure 1b), which occurs due to the lipids being compressed in the plane of the membrane, leading to freezing of the lipid tails. We have extensively investigated this transition in single lipid systems in the presence of sugars, controlling the moisture contents by equilibration with various relative humidities [35–38]. Depending on the phospholipid used, other membrane lipid phases may also exist at low hydration. These include the inverse hexagonal phase (Figure 1c) and the ribbon phase (Figure 1d). Such non-bilayer phases have been studied extensively [39–46] and have been shown to be important in freezing and dehydration damage in biological systems [6,47–50]: the non-lamellar nature of these phases means that cell membranes, which undergo such transitions, can no longer function as semipermeable barriers between the cell and its environment.

The measurements reported here will cover a number of these phases and highlight the complementary type of information that can be gained from a range of scattering techniques. The data presented has a particular emphasis on the localization and quantification of the sugar concentrations close to the lipid head groups. However, the techniques described can be applied to the study of any small molecules, which may interact with membranes and affect membrane structure.

## 2. Scattering Techniques

In order to test quantitative models of the interactions of membranes with sugars [4,8,36,37], membrane structural parameters need to be measured using scattering techniques. These techniques have been applied with great effect to understand the phase behavior and structure of phospholipids on the nanoscale [51–55]. Not only are they diagnostic of the phase of the lipids, but they are able to extract structural details relating to the shape and spatial relationships between coexisting phases. In our case, they provide two very important structural parameters: the average chain-chain lateral spacing (which can be used to estimate the average headgroup spacing *d*_h_), and the bilayer repeat spacing d, which can be used to estimate *d*_w_ and *d*_B_ (Figure 1a). This information can be supplemented by contrast variation SANS; this is a low resolution quantitative technique, which, combined with a good knowledge of the density, isotopic makeup and volume fraction of each component in the sample, allows us to understand the length-scale and nature of heterogeneities quantitatively.

### 2.1. X-Ray Scattering

Experimental measurements are made both on synchrotron [37] and lab-based X-ray sources [39]. The basic principle of the experiments is the same, where the scattered intensity is measured as a function of scattering angle relative to the incident beam [56]. In the cases discussed here, the scattered intensity is measured on an area detector behind the sample. As an issue of scattering formalism and its theoretical treatment and also so that measurements made with different wavelengths of scattering radiation may be compared, the scattered intensity is expressed in terms of the scattering vector, *q*, which represents the modulus of the change in momentum of the scattered radiation:

(1) $$q=\frac{4\pi }{\lambda }sin\theta$$

where λ is the wavelength of the scattered radiation and 2θ is the scattering angle relative to the incoming beam. The features, which are most prominent in the X-ray scattering patterns, are the peaks. The angular position of these peaks is indicative of a periodic spacing within the sample, and they may be used to assign the lipid space group and measure periodic spacings within the sample (e.g., Figure 1). The characteristic distances, *d*, of the spacing may be calculated simply using the Bragg equation and the angular position of a first order diffraction peak:

(2) $$n\lambda =2d.sin\theta_{peak}$$

where *n* is a positive integral number and is the order of the reflection (*n* = 1 for first order) and λ the wavelength of the radiation. For the first order scattering peak, this can be rewritten as:

(3) $$d=\frac{2\pi }{q_{peak}}$$

**Figure 2:**
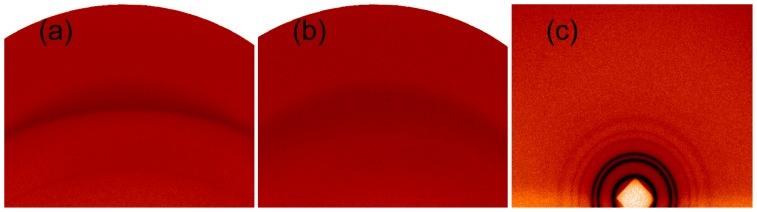
Small angle X-ray scattering (SAXS) patterns for DOPC (1,2-dioleoyl-*sn*-glycero-3-phosphocholine): (**a**) gel phase at −33 °C; (**b**) fluid phase at 2.6 °C; and (**c**) inverse hexagonal phase at 36 °C. In comparison with the fluid phase (**b**), the gel phase (**a**) has an extra reflection and stronger reflections. The inverse hexagonal phase (**c**) has more reflections at small angles.

The samples considered here are “powder” type samples—*i.e.*, they consist of many stacks of lipid bilayers oriented randomly, yielding isotropic scattering with respect to the incident beam [57], as shown by the two-dimensional (powder) patterns, examples of which are shown in Figure 2. Figure 2a,b shows, respectively, a gel phase and a fluid phase. The gel phase (Figure 2a) is indicated by the extra reflections, as well as the strength of the reflections relative to those in the fluid phase (Figure 2b). This is due to the increased order in the packing of the lipid chains in the gel phase relative to the fluid phase. Figure 2c shows the scattering pattern on an image detector from an inverse hexagonal phase, indicated by the non-linear spacing of the reflections, which index to and indicate the important cell dimensions of hexagonal packing (Figure 1c).

**Figure 3:**
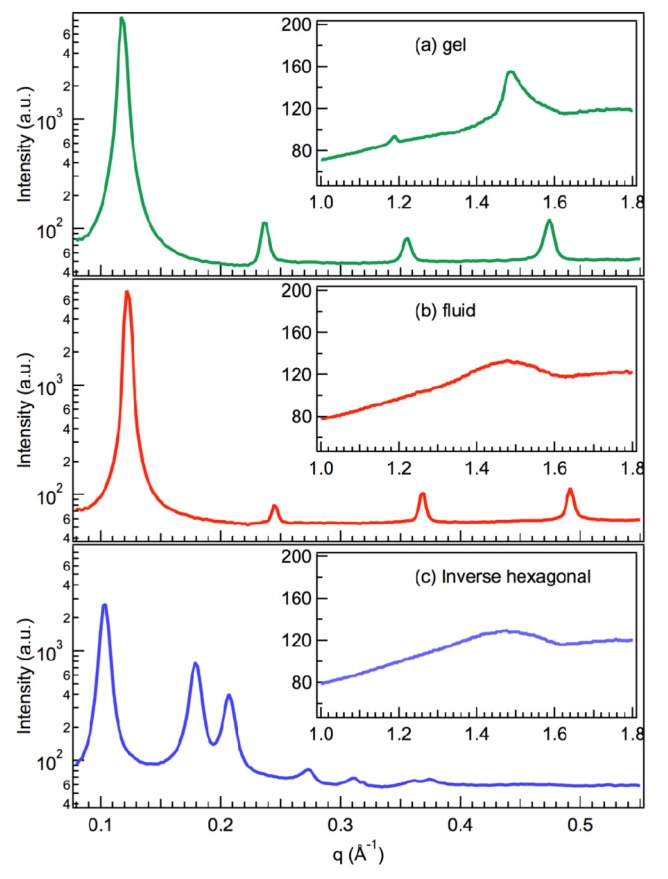
Radial averages of the data in Figure 2, plotted as intensity *vs. q*. The positions of the peaks determine the phase and relevant structural parameters. For the (**a**) gel and (**b**) fluid phases, the primary peak at low *q* determines the repeat spacing, d (Figure 1a). As both samples are lamellar, the higher order peaks are the second, third, fourth and fifth order reflections of this primary repeat spacing. For the (**c**) inverse hexagonal phase, the reflections yield d_11_ and d_10_. The wide-angle peaks (shown in the insets) yield the average chain-chain separation. The sharp peak in (**a**) is indicative of the ordered gel phase, while the other two samples have chains in the fluid configuration, giving a broad peak.

The pinhole scattering data is radially symmetric, and knowing the experimental geometry and wavelength of the scattered radiation, the data may be radially averaged and plotted as intensity *versus* the scattering vector, *q*, as shown in Figure 3. Each peak corresponds to a different order of Bragg scattering, which allows the determination of the relevant structural parameters for each phase. The WAXS radial averages are shown as insets in Figure 3. The broad peaks for the fluid and inverse hexagonal phases (Figure 3b,c) indicate that the chains are in the fluid configuration, while the sharper peak in Figure 3a is indicative of the tighter packing in the gel phase.

On modern synchrotron X-ray scattering beam lines [58], we are able to measure the positions of these peaks to good precision on timescales of the order seconds [37,38], as well as using the tunable nature of the X-ray radiation to access different regions of reciprocal/*q*-space. This approach allows us to measure the inter-bilayer spacing and the inter-lipid packing in bilayers (Figure 3) using a single instrument configuration for a large number of samples and temperatures, as well as allowing a study of the kinetics of the transitions with or without sugars [38]. Therefore, many measurements would be impractical using lab-based X-rays sources, and kinetic measurements would be impossible.

The determination of the structural parameters for each phase at a range of hydrations and temperatures enables us to link the HFE theory of inter-membrane interactions [4,26] to the structural changes caused by lateral compression in the membrane [37]. Additional information in the shape and relative intensities of higher order peaks, *n* > 1, allows the reconstruction of the electron density profiles. Calculations of the electron density profile found that the electron density in the head group region is not altered by the presence of sugars in the aqueous phase [40]. This finding reinforces the conclusion that sugars are not preferentially located at the lipid head groups in partially dried samples.

### 2.2. Small Angle Neutron Scattering

The technique of contrast variation SANS has particular power in this scientific problem. Although the technique inherently provides lower resolution than SAXS, its main advantage in this case is that the measurement provides quantitative information more easily than SAXS, but also instruments are easily optimized for measurements over an extended *q*-range with a range of configurations/sample to detector distances. In common X-ray scattering measurements, the scattered intensity is measured as a function of small angles around the direction of the primary beam of neutrons, and the formalism is the same. For neutrons, however, rather than the heterogeneities being due to variations in electron density, as is the case for X-ray scattering, heterogeneities are due to variations in nuclear properties of the constituents—*i.e.*, the scattering length density (SLD). Isotopic substitution, generally of deuterium for hydrogen nuclei, allows us to vary the scattering contribution of various sample components without changing the physics or chemistry of the system in an appreciable way. This isotopic substitution, or contrast variation, is a generic means to improve the information content of the low-resolution SANS technique [59,60]. In the case of crystallographic reconstruction of unit cells, it may also be used to determine the phasing of the Fourier terms in the reconstruction of a unit cell [54]; however, conventional pinhole SANS instruments, which are optimized for neutron flux, have a larger spread of wavelengths and a smaller dynamic *q*-range [61] compared to typical SAXS beam lines [58]; so, the resolution is lower.

We have extended the work of Demé and Zemb [57] to include a range of sugars, hydrations and lipids. Regardless of the lamellar system used, we found that the presence of sugars leads to two aqueous phases in equilibrium with each other, but with quite different concentrations of sugar: one aqueous phase between the bilayers in the lamellae and another, which does not contribute measurably to the SANS signal, in a bulk phase [35].

**Figure 4:**
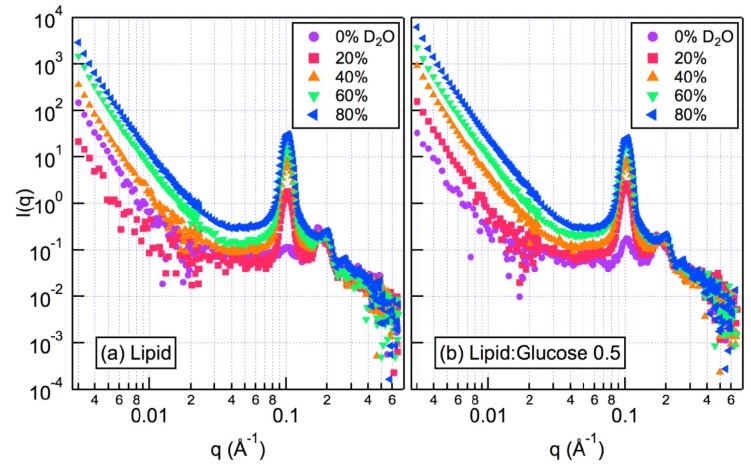
Radially averaged small angle neutron scattering (SANS) data from (**a**) DOPE and (**b**) 0.5:1 glucose:DOPE for varying amounts of D_2_O. The data show the typical form of a low *q* linear region and a peak due to the (1,0) plane of the H_II_ phase at s higher *q*.

We have also applied contrast variation to non-lamellar lipids, hydrating with varying ratios of H_2_O:D_2_O. For SANS experiments, deuterated glucose is used in order to enhance the neutron contrast—in our case, D_6_-glucose. Figure 4 shows an example of such a contrast variation series for 1,2-dioleoyl-*sn*-glycero-3-phosphoethanolamine (DOPE) in excess water at 45 °C, without (Figure 4a) and with (Figure 4b) deuterated glucose. Each experiment was conducted with five different H_2_O:D_2_O ratios—changing the ratio of H_2_O to D_2_O in the aqueous phase changes the relative scattering power (contrast) between the aqueous phase and the membrane phase. In the sample containing only water and lipid, the contrast is due to the differences in scattering light density (SLD) of the lipid and the aqueous phases. If the sample contains sugar, the scattering length density of the aqueous will also contain contributions from atoms other than those that reflect the composition of the solvent, *i.e.*, the exchangeable hydrogens attached to hydroxyl groups. The scattering curves on a log-log plot typically consist of a linear region, where the slope is close to −4, and one or more peaks, which are equivalent to Bragg peaks found by x-rays, but broadened by the instrumental convolution of the SANS instrument [57].

**Figure 5:**
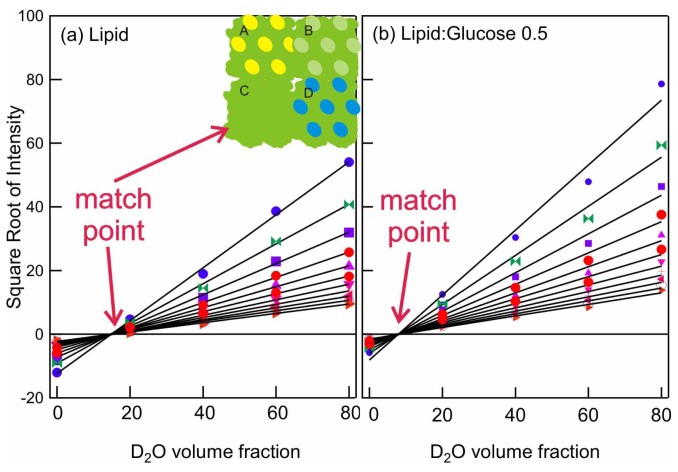
Square root of intensity *vs.* D_2_O volume fraction for the data in Figure 4. A schematic representation where the scattered intensity is proportional to the contrast between the two phases is shown in the inset. In this case, the scattering is due to the contrast, (or difference in scattering length density)^2^, between the aqueous phase (various ratios of H_2_O:D_2_O:D_6_-glucose) and the lipid phase.

In order to determine the concentrations and locations of the sugars, the data in Figure 4 are analyzed by taking the square root of the scattered intensity at several different *q*-values, which lie in the linear region of the log-log plot. These values are then plotted as functions of theD_2_O volume fraction, as shown in Figure 5. Each line represents the intensity values found at a single *q*-value (a vertical line through the scattering curves in Figure 4). The variation of intensity at a particular *q*-value from each data set with a different H_2_O:D_2_O ratio may be described by a quadratic equation, and a best-fit quadratic equation can be determined for each *q*-value. Where these lines cross, the axis is the point of zero scattered intensity, called the match point: this is the point at which there is no contrast between lipid and the aqueous water channels in the H_II_ phase, as shown schematically in the inset to Figure 5a. The minimum contrast, where the lines pass through zero, is represented by Schematic C. Since the isotopic ratio of water and the match point with a pure lipid phase are known, one can calculate the shift from the pure lipid value and, thus, the contribution of the D_6_-glucose in the channels to the match point. The position of the match point in the presence of glucose (b) allows the calculation of the sugar concentration in the water channels of the H_II_ phase. As shown in the figures, the lines all cross zero at the same point—this tells us that the match point is *q*-independent in the low *q* region, which is vital for the analysis to be valid [57].

The match point is different for the pure lipid (Figure 5a) and the lipid with glucose (Figure 5b), since the composition of the solvent in the latter case has been altered by the D_6_-glucose. Thus, from this data set, it is possible to calculate the concentrations of sugar in the aqueous water channels of the H_II_ phase. Calculations reveal that the glucose concentration in the aqueous channels is lower than that in the bulk phase. This result demonstrates that sugars are partially excluded from the H_II_ phase water channels, implying that there are no dominant sugar-head group interactions and lending support to the HFE for the protective role of sugars during dehydration.

## 3. Discussion and Conclusions

Access to large scale facilities, in particular, synchrotron and neutron small angle scattering, has allowed us to quantify factors relevant to the dehydration protection and cryo-protection of membranes by small solutes, specifically the distance between lipid membranes and the spacing between lipid molecules packed in the membrane. Synchrotron X-ray scattering techniques provide a rapid method for measuring important structural parameters and allow us to make measurements on more samples and conditions than would be possible using lab-based X-ray equipment. The resulting measurements have validated the hydration forces explanation (HFE) by directly relating the separation between lipid bilayers and the separation between head groups during the same measurement [37]. Contrast variation SANS allows the link between the sugar concentration in the lipid phase (lamellar or H_II_) to precise structural information from X-ray scattering.

Contrast variation SANS measurements on model systems indicates the exclusion of sugar molecules from between bilayers. While it is clear that this is an excluded volume effect, since larger molecules are excluded more effectively than smaller molecules [62], the quantification of this solute exclusion was not previously possible. SANS measurements take longer than synchrotron measurements (e.g., on the order of tens of hours for the contrast variation series shown in Figures 4 and 5, whereas a single synchrotron measurements takes on the order of seconds), and while improvements in the neutron flux of modern SANS instruments may provide improvements on this situation, it will remain a low throughput technique. It is therefore very important to conduct complementary measurements using laboratory-based equipment to identify regions of interest prior to attempting SANS measurements. For example, for the samples studied here, previous differential scanning calorimetry measurements have shown that the effects of sugars on the phase transition temperature saturate with increasing concentration [36], reducing the number of samples, which need to be studied using SANS.

Our investigations of the partitioning of sugars in lipid systems have mostly concentrated on the lamellar systems, in particular, the fluid to gel phase transition. However, it is known that non-lamellar phases, such as the inverse hexagonal phase [41,42], are a critical component of dehydration and freezing damage [63]. Recently, we have shown how the techniques described above may be extended to studying the effects of sugars on non-lamellar phases, in particular, the inverse hexagonal and ribbon phases (Figure 1c,d) [39,40]. These studies showed that the addition of glucose to a fully hydrated DOPE inverse hexagonal phase (Figures 2c and 3c) had no significant effect on the structure of the phase and that glucose was (partially) excluded, similar to the results for the lamellar phases. However, these results also showed that the presence of glucose enhances the formation of the inverse hexagonal phase, which is in contrast with the observed ability of sugars to limit damage to biological cells during dehydration. These results are currently undergoing further investigation.

In biological systems, transitions to non-lamellar phases would clearly lead to a loss in the continuity of the barrier properties of the cell membrane. Thus, despite the challenges posed by these systems, X-ray diffraction and contrast variation SANS should provide a means for relating the important structural parameters to the partitioning of aqueous sugar molecules. Further experiments along these lines are currently underway.

The lower limit of hydration explored by the contrast variation technique, a sample equilibrated with 32% relative humidity, is greater than that experienced by many real membranes in extremely dry conditions. SANS measurements on materials at such low hydration levels are limited by the low signal, which is present at low moisture contents. One way of improving the signal to noise in SANS data from such samples is by deuteration of the lipid phase. Gains in signal will be made due to the enhanced contrast between aqueous and lipid phases, as well as the lower incoherent signal from the lipid phase [64], and this will allow us to obtain good quality measurements for systems at low hydration levels. Deuteration facilities are now becoming recognized as an integral tool in neutron scattering studies of biological systems (e.g., Institute Laue Langevin, [65]; Australian Nuclear Science and Technology, organization, [66]; and the Center for Structural Molecular Biology, Oak Ridge National Laboratory, [67]). Deuteration of lipid phases will also provide an invaluable tool in the phasing problem for the reconstruction of scattering length densities of orientated membrane systems and allow for higher resolution studies of the sugar concentration profile between lipid head groups [68]. These studies will provide a valuable complement to the electron density reconstructions of isotropic phases.

## 4. Materials and Methods

### 4.1. Small Angle X-Ray Scattering

SAXS/WAXS experiments were conducted at the Australian Synchrotron SAXS/WAXS beamline with λ = 0.827 Å and a sample to detector distance of 548 mm. Diffraction patterns were recorded on a 2D Dectris Pilatus 1M detector over a range of scattering vectors from 0.073 to 1.96 Å^−1^, covering the length scales of interest for the primary repeat distance and the wide angle reflection. Exposure times were 1.9 s. Samples were inserted into 1.5 mm quartz capillaries and sealed with epoxy resin. The pinhole scattering data is radially symmetric and, together with the experimental geometry and wavelength of the scattered radiation, may be used to produce the radial average shown in Figure 3. The positions of the peaks may be used to measure the important inter lamellar and lipid packing spacings shown in Figure 1.

### 4.2. Small Angle Neutron Scattering

SANS measurements were performed on the QUOKKA beamline at the Bragg Institute (ANSTO, Australia) [61]. Samples were mounted in 0.2 mm path length Hellma cells. The incident neutron wavelength was 5 Å, with a resolution of 10% Δλ/λ (FWHM). Measurements were made at sample to detector distances of 20.17 m, 4.52 m and 1.24 m, giving a combined *q*-range of 3.000 × 10^−3^ to 6.645 × 10^−1^ Å^−1^. Measurement count times at each distance were 30 min, 20 min and 10 min, respectively. Sample cells were sealed with lab wrap and positioned on a multi-sample changer adjacent to a cadmium mask with a 1 cm diameter cutout. Sample temperature was controlled using a circulating water bath. Measurements were made at 45 °C and were equilibrated for >1 h prior to measurement. Further instrument specifications and details of the data reduction can be found online [69].

Neutrons were detected on a two-dimensional position sensitive detector. After correction for the detector response, the two-dimensional scattering patterns were checked to be isotropic at the three different camera lengths. Scattering patterns were normalized to the empty beam flux and sample thickness and appropriate backgrounds due to the empty cell and the isotropic incoherent signal, due mainly to ^1^H in the sample, subtracted.

### 4.3. Sample Preparation

Samples in all cases are prepared by exposing known molar ratios of lipids and sugars to a known humidity or by adding water gravimetrically. With increasing water content, d-spacings will increase, the moisture content at which the d-spacing does not expand further [*cf* Equations (2) and (3)] and water partitions only into a bulk excess water phase is termed excess hydration. Further details of preparing samples and their equilibration at different hydrations are given elsewhere [37]. For SANS measurements, the ratio of D_2_O:H_2_O was varied, and the samples packed to uniform density in quartz cells of a 200 μm path length with care taken not to shear the samples. Oriented structures will produce anisotropic scattering patterns and make quantitative analysis difficult. X-ray scattering measurements were made on samples packed into thin-walled quartz capillaries of 1.5 mm diameter.

## References

[1] Hoffmann E.K., Simonsen L.O. (1989). Membrane mechanisms in volume and pH regulation in vertebrate cells. Physiol. Rev.

[2] Yeo A.R., Flowers T.J. (2008). Plant Solute Transport.

[3] Maurel C. (1997). Aquaporins and water permeability of plant membranes. Annu. Rev. Plant Physiol. Plant Molec. Biol.

[4] Wolfe J., Bryant G. (1999). Freezing, drying, and/or vitrification of membrane-solute-water systems. Cryobiology.

[5] Benga G. (2009). Water channel proteins (Later Called Aquaporins) and relatives: Past, present, and future. IUBMB Life.

[6] Koster K.L., Bryant G, Chen T.H.H., Uemura M., Fujikawa S. (2005). Dehydration in Model Membranes and Protoplasts: Contrasting Effects at Low, Intermediate and High Hydrations. Cold Hardiness in Plants: Molecular Genetics, Cell Biology and Physiology.

[7] Steponkus P.L. (1984). Role of the plasma membrane in freezing injury and cold acclimation. Annu. Rev. Plant Physiol. Plant Molec. Biol.

[8] Andersen H.D., Wang C., Arleth L., Peters G.H., Westh P. (2011). Reconciliation of opposing views on membrane-sugar interactions. Proc. Natl. Acad. Sci. USA.

[9] Westh P. (2008). Glucose, sucrose and trehalose are partially excluded from the interface of hydrated DMPC bilayers. Phys. Chem. Chem. Phys.

[10] Billi D., Potts M. (2002). Life and death of dried prokaryotes. Res. Microbiol.

[11] Koster K.L., Leopold A.C. (1988). Sugars and desiccation tolerance in seeds. Plant Physiology.

[12] Storey K.B., Storey J.M. (1996). Natural freezing survival in animals. Annu. Rev. Ecol. Syst.

[13] Uemura M., Warren G., Steponkus P.L. (2003). Freezing sensitivity in the sfr4 mutant of *Arabidopsis* is due to low sugar content and is manifested by loss of osmotic responsiveness. Plant Physiol.

[14] Crowe L.M., Crowe J.H., Rudolph A., Womersley C., Appel L. (1985). Preservation of freeze-dried liposomes by trehalose. Arch. Biochem. Biophys.

[15] Strauss G., Schurtenberger P., Hauser H. (1986). The interaction of saccharides with lipid bilayer vesicles—Stabilization during freeze-thawing and freeze-drying. Biochim. Biophys. Acta.

[16] Crowe J.H., Crowe L.M., Chapman D. (1984). Preservation of membranes in anhydrobiotic organisms—The role of trehalose. Science.

[17] Crowe J.H., Crowe L.M., Oliver A.E., Tsvetkova N., Wolkers W., Tablin F. (2001). The trehalose myth revisited: Introduction to a symposium on stabilization of cells in the dry state. Cryobiology.

[18] Potts M. (1994). Desiccation tolerance of prokaryotes. Microbiol. Rev.

[19] Bryant G., Koster K.L., Wolfe J. (2001). Membrane behaviour in seeds and other systems at low water content: The various effects of solutes. Seed Sci. Res.

[20] Koster K.L., Webb M.S., Bryant G., Lynch D.V. (1994). Interactions between soluble sugars and POPC (1-palmitoyl-2-oleoylphosphatidylcholine) during dehydration-vitrification of sugars alters the phase-behavior of the phospholipid. BBA-Biomembranes.

[21] Sun W.Q., Leopold A.C., Crowe L.M., Crowe J.H. (1996). Stability of dry liposomes in sugar glasses. Biophys. J.

[22] Koster K.L., Lei Y.P., Anderson M., Martin S., Bryant G. (2000). Effects of vitrified and nonvitrified sugars on phosphatidylcholine fluid-to-gel phase transitions. Biophys. J.

[23] Wolfe J. (1987). Lateral stresses in membranes at low water potential. Aust. J. Plant Physiol.

[24] Bryant G., Wolfe J. (1988). Lateral phase separations in lipid lamellar phases at low hydration. Cryobiology.

[25] Bryant G., Wolfe J. (1989). Can hydration forces induce lateral phase separations in lamellar phases. Euro. Biophys. J.

[26] Bryant G., Wolfe J. (1992). Interfacial forces in cryobiology and anhydrobiology. Cryo-Letters.

[27] Horn R.G. (1984). Direct measurement of the force between 2 lipid bilayers and observation of their fusion. Biochim. Biophys. Acta.

[28] Lis L.J., McAlister M., Fuller N., Rand R.P., Parsegian V.A. (1982). Interactions between neutral phospholipid bilayer membranes. Biophys. J.

[29] Rand R.P., Parsegian V.A. (1989). Hydration forces between phospholipid bilayers. Biochim. Biophys. Acta.

[30] Lis L.J., McAlister M., Fuller N., Rand R.P., Parsegian V.A. (1982). Measurement of the lateral compressibility of several phospholipid-bilayers. Biophys. J.

[31] Pincet F., Perez E., Wolfe J. (1994). Do trehalose and dimethyl sulfoxide affect intermembrane forces?. Cryobiology.

[32] Bryant G., Pope J.M., Wolfe J. (1992). Low hydration phase properties of phospholipid mixtures—Evidence for dehydration-induced fluid-fluid separations. Euro. Biophys. J.

[33] Tardieu A., Luzzati V., Reman F.C. (1973). Structure and polymorphism of the hydrocarbon chains of lipids: A study of lecithin-water phases. J. Mol. Biol.

[34] Luzzati V, Chapman, D. (1968). X-ray Diffraction Studies of Lipid-Water Systems. Biological Membranes: Physical Fact and Function.

[35] Lenné T., Bryant G., Garvey C.J., Keiderling U., Koster K.L. (2006). Location of sugars in multilamellar membranes at low hydration. Physica B.

[36] Lenné T., Bryant G., Holcomb R., Koster K.L. (2007). How much solute is needed to inhibit the fluid to gel membrane phase transition at low hydration?. BBA-Biomembranes.

[37] Lenné T., Garvey C.J., Koster K.L., Bryant G. (2009). Effects of sugars on lipid bilayers during dehydration: SAXS/WAXS measurements and quantitative model. J. Phys. Chem.

[38] Lenné T., Garvey C.J., Koster K.L., Bryant G. (2010). Kinetics of the lamellar gel-fluid transition in phosphatidylcholine membranes in the presence of sugars. Chem. Phys. Lipids.

[39] Kent B., Garvey C.J., Cookson D., Bryant G. (2009). The inverse hexagonal—Inverse ribbon—Lamellar gel phase transition sequence in low hydration DOPC:DOPE phospholipid mixtures. Chem. Phys. Lipids.

[40] Kent B., Garvey C.J., Lenne T., Porcar L., Garamus V.M., Bryant G. (2010). Measurement of glucose exclusion from the fully hydrated DOPE inverse hexagonal phase. Soft Matter.

[41] Pohle W., Selle C. (1996). Fourier-transform infrared spectroscopic evidence for a novel lyotropic phase transition occurring in dioleoylphosphatidylethanolamine. Chem. Phys. Lipids.

[42] Pohle W., Selle C., Gauger D.R., Brandenburg K. (2001). Lyotropic phase transitions in phospholipids as evidenced by small-angle synchrotron X-ray scattering. J. Biomol. Struct. Dyn.

[43] Gruner S.M. (1989). Stability of lyotropic phases with curved interfaces. J. Phys. Chem.

[44] Tate M.W., Gruner S.M. (1987). Lipid polymorphism of mixtures of dioleoylphosphatidylethanolamine and saturated and monounsaturated phosphatidylcholines of various chain lengths. Biochemistry.

[45] Seddon J.M. (1990). Structure of the inverted hexagonal (HII) phase, and non-lamellar phase transitions of lipids. BBA-Rev. Biomebranes.

[46] Epand R.M. (1996). Functional roles of non-lamellar forming lipids. Chem. Phys. Lipids.

[47] Pearce R.S. (1985). A freeze-fracture study of the cell-membranes of wheat adapted to extracellular freezing and to growth at low-temperatures. J. Exp. Bot.

[48] Pearce R.S. (1985). The membranes of slowly drought-stressed wheat seedlings—A freeze-fracture study. Planta.

[49] Platt-Aloia K.A., Aloia, R.C., Curtin, C.C., Gordon, L.M. (1988). Freeze-Fractue Evidence of Stressed-Induced Phase Separations in Plant Cell Membranes. Physiological Regulation of Membrane Fluidity, Advances in Membrane Fluidity.

[50] Uemura M., Joseph R.A., Steponkus P.L. (1995). Cold-acclimation of arabidopsis-thaliana—Effect on plasma-membrane lipid-composition and freeze-induced lesions. Plant Physiol.

[51] Caffrey M, LEOPOLD A.C. (1986). X-Ray Diffraction as a Technique for Studying the Mesomorphic Phase Properties of Lipids. Membranes, Metabolism, and Dry Organisms; Conference on Anhydrous Biology, Bellagio, Italy.

[52] Caffrey M. (1985). Kinetics and mechanism of the lamellar gel lamellar liquid-crystal and lamellar inverted hexagonal phase-transition in phosphatidylethanolamine—A real-time X-ray-diffraction study using synchrotron radiation. Biochemistry.

[53] Kucerka N., Nagle J.F., Sachs J.N., Feller S.E., Pencer J., Jackson A., Katsaras J. (2008). Lipid bilayer structure determined by the simultaneous analysis of neutron and X-ray scattering data. Biophys. J.

[54] Nagle J.F., Tristram-Nagle S. (2000). Structure of lipid bilayers. BBA-Rev. Biomebranes.

[55] Ding L., Liu W., Wang W., Glinka C.J., Worcester D.L., Yang L., Huang H.W. (2004). Diffraction techniques for nonlamellar phases of phospholipids. Langmuir.

[56] Warren B.E. (1969). X-Ray Diffraction.

[57] Deme B., Zemb T. (2000). Measurement of sugar depletion from uncharged lamellar phases by SANS contrast variation. J. Appl. Crsytallogr.

[58] Cookson D., Kirby N., Knott R., Lee M., Schultz D. (2006). Strategies for data collection and calibration with a pinhole-geometry SAXS instrument on a synchrotron beamline. J. Synchrotron Radiat.

[59] Svergun D.I., Koch M.H.J. (2003). Small-angle scattering studies of biological macromolecules in solution. Rep. Prog. Phys.

[60] Pan J.J., Heberle F.A., Tristram-Nagle S., Szymanski M., Koepfinger M., Katsaras J., Kucerka N. (2012). Molecular structures of fluid phase phosphatidylglycerol bilayers as determined by small angle neutron and X-ray scattering. Biochim. Biophys. Acta-Biomembr.

[61] Gilbert E.P., Schulz J.C., Noakes T.J. (2006). Quokka. Physica B.

[62] Koster K.L., Maddocks K.J., Bryant G. (2003). Exclusion of maltodextrins from phosphatidylcholine multilayers during dehydration: Effects on membrane phase behaviour. Eur. Biophys. J.

[63] Gordon-Kamm W.J., Steponkus P.L. (1984). Lamellar-to-hexagonal II phase-transitions in the plasma-membrane of isolated protoplasts after freeze-induced dehydration. Proc. Natl. Acad. Sci.Biol.

[64] Jacrot B. (1976). Study of biological structures by neutron-scattering from solution. Rep. Prog. Phys.

[65] Institute Laue Langevin.

[66] Australian Nuclear Science and Technology, organization.

[67] Center for Structural Molecular Biology, Oak Ridge National Laboratory http://www.csmb.ornl.gov/bdl/.

[68] Wiener M.C., White S.H. (1991). Fluid bilayer structure determination by the combined use of X-ray and neutron-diffraction. 1. Fluid bilayer models and the limits of resolution. Biophys. J.

[69] Quokka—Small-Angle Neutron Scattering.

